# Example for process validation in biobanking: Fit for purpose testing of a cryopreservation method without isopentane

**DOI:** 10.3389/fmolb.2022.876670

**Published:** 2022-09-30

**Authors:** Monika Wieser, Stefanie Burger, Reinhard Ertl, Stefan Kummer, Melanie Stargardt, Ingrid Walter

**Affiliations:** VetCore Facility for Research, University of Veterinary Medicine Vienna, Vienna, Austria

**Keywords:** biobanking, process validation, snap-freezing, tissue, morphology, RNA integrity

## Abstract

**Background:** The freezing process of tissue samples is crucial for the preservation of morphological and molecular features. Several biobanking guidelines describe freezing techniques for optimal outcomes. As the Vetbiobank standard freezing protocol does not comply with those recommendations in detail, a process validation was performed to demonstrate that samples are suitable for downstream applications. Here we give a formal example of a process validation in the biobanking setting, as required by the biobanking guideline ISO 20387 (2018).

**Methods:** Three different freezing protocols, freezing in liquid nitrogen, freezing *via* isopentane precooled on dry ice and freezing *via* liquid nitrogen vapor, were assessed based on morphological integrity of mouse liver and muscle tissue samples. Samples were either frozen in cryotubes (without Optimal Cutting Temperature compound, OCT) or in cryomolds (with OCT). The protocol providing the best results was validated for reproducibility and robustness in terms of defined acceptance criteria for morphological evaluability, A260/A280 ratio, and RNA integrity number values (RIN). In addition, performance tests were run by gene expression analyzes of selected, tissue specific biomarkers to confirm that processed samples are fit for purpose.

**Results:** From the three applied freezing protocols, freezing in liquid nitrogen generated best results. Reproducibility acceptance criteria were met for both, morphological integrity and RNA quality. The freezing method was robust for the tested tissue types and the application of OCT, with exception of liver tissue, where it led to a significant decrease of the RIN value. Gene expression analyzes showed good comparability of results regardless of the applied freezing method.

**Conclusion:** Freezing of tissue samples in liquid nitrogen provides samples of adequate quality for subsequent RNA investigations. A negative impact of OCT on the RIN value of liver samples was observed, which was independent from the applied freezing protocol and showed no impact on subsequent gene expression analysis.

## Introduction

The main attempt of tissue biobanks is the optimal preservation of samples to enable the application of a wide panel of downstream analysis methods. Generally, two methods for tissue preservation predominate in biobanking: On the one hand, tissues are fixed in formaldehyde, dehydrated and embedded in paraffin wax (FFPE). This has been the most common method for pathologic laboratories for decades up until now because of excellent structural tissue preservation, convenient handling and low-cost storing of paraffin blocks at room temperature. Therefore, FFPE-tissue samples are the current gold standard for histo-pathological analyzes and antibody-based methods on histological sections.

On the other hand, tissues are cryopreserved. Frozen tissues are favored for molecular testing because proteins and nucleic acids are preserved in a native state, not being modified and degraded by a cross-linking agent in contrast to FFPE-tissues (Micke et al., 2006; Charde et al., 2014; Shabihkhani et al., 2014; Galissier et al., 2016). However, freezing might have a negative effect on the structural integrity of the tissue. Since morphological evaluation is required to verify the sample material, several guidelines and publications provide detailed recommendations for freezing tissue samples to reduce the adverse effects of freezing on cell integrity.

For tissue banking, where the specimen *in vivo* state (e.g., morphology, gene expression) but not viability of cells needs to be conserved, three main techniques have become accepted standard procedures: 1) freezing in liquid nitrogen or dry ice, 2) freezing using a precooled cryoconductor like isopentane or isobutene and 3) freezing in nitrogen vapor. Freezing in liquid nitrogen is the easiest and fastest method for snap-freezing, but the freezing process itself is hampered by the Leidenfrost effect. This effect leads to the formation of a vapor layer around warm surfaces, which inhibits direct contact to liquid nitrogen or dry ice and therefore slows down the freezing process (Baker et al., 2013). As rapid freezing is considered to be crucial for the preservation of morphological and molecular features, most biobanking best practice guidelines and/or standard protocols recommend the use of a freezing medium for snap-freezing. The IARC technical publication No44 (Mendy et al., 2017) and the General TuBaFrost SOP (Mager et al., 2007) strictly advise the use of isopentane for freezing, while the ISO 20184 (2018), the Australasian Biospecimen Network (2007) and the Molecular Medicine Irland (Doran et al., 2010) favor the use of isopentane but specify direct freezing in liquid nitrogen as valid alternative. The ISBER Best Practices (2018) and the Canadian Tissue Repository Network (2020) describe both methods without emphasizing one. In contrast to the above-mentioned methods, the NCI Best Practices (2016) alternatively favors the freezing in liquid nitrogen vapor to overcome the freezing delay caused by the Leidenfrost effect. However, snap-freezing by freezing in liquid nitrogen or *via* isopentane are included as accepted alternatives. Additionally, the use of Optimal Cutting Temperature compound (OCT) is recommended by the ISBER Best Practices (2018), the Canadian Tissue Repository Network (2020), the IARC technical publication No44 (Mendy et al., 2017), the General TuBaFrost SOP (Mager et al., 2007), the NCI Best Practices (2016), the Australasian Biospecimen Network (2007) and the Molecular Medicine Irland (Doran et al., 2010) if good preservation of cellular structure is required. As listed above, different snap-freezing techniques are in place for cryopreservation and different biobanking guidelines favor different methods.

In the present study, we want to give a formal example for the performance of a process validation, which is requested by the new biobanking guideline ISO 20387 (2018), if the performed processing method does not comply in detail with published standard processing methods. The study was divided into three parts. In the first part, the optimization part, we compared different freezing methods. In the second part, the validation part, we chose the freezing method with the best results regarding morphological criteria and tested it for reproducibility and robustness in terms of quality markers, according to recommendations of the ISO 21899 (2020), and formerly published processing method validations (Ammerlaan et al., 2014a; Ammerlaan et al., 2014b; Hamot et al., 2015; Mathay et al., 2016; Neuberger-Castillo et al., 2020). In the third part, performance was tested by RT-qPCR analyzes of defined biomarkers to demonstrate that RNAs extracted from these frozen samples are fit for purpose.

We wanted to investigate whether snap freezing *via* isopentane, the common standard procedure of most pathology departments, is inevitable to enable morphological assessment of frozen tissue samples. We chose liver and muscle tissue for our study, because liver samples are described to show good morphological integrity with and without the use of isopentane, while for muscle tissues the use of isopentane is strongly recommended to avoid freezing artefacts (Meng et al., 2014; Kap et al., 2015).

## Material and methods

### Sample collection plan and experimental design

Liver and muscle tissues of three C57BL/6 mice were dissected and snap frozen immediately after animal euthanasia (Table 1). An initial protocol optimization was performed with respect to the freezing technique with freezing in liquid nitrogen versus freezing *via* isopentane precooled on dry ice versus freezing in the vapor of liquid nitrogen using the FluidX (now Brooks Life Science) CryoPod™-Carrier. The Cryopod Carrier was used outside its dedicated application, namely the transport of cryopreserved tissue, as its construction meets the experimental setup of the NCI Best Practices (2016), for snap freezing in liquid nitrogen vapor. All samples were prepared in duplicates with and without OCT (Sakura), samples were either frozen in cryotubes (without OCT) or in cryomolds (with OCT). In the first part of the study, the optimization part, different freezing protocols were assessed for morphological integrity. Tissue morphology must be preserved in a quality that allows recognition of tissue type and tissue-specific cells and compartments e.g., tissue type, cell nuclei, tumor cells, and necrosis. In order to meet this requirement, haematoxylin and eosin (H&E) stains were considered to be evaluable when more than 50% of the analyzed areas were staged to be “very good”, “good” or at least “average”. Six H&E stained sections were assessed per freezing method and tissue type. For evaluation, minimum values of evaluable areas of the six corresponding slides were taken into account, in order to ensure morphological evaluation.

**TABLE 1:** Sample collection and experimental plan.

Freezing protocol		Direct freezing *via* liquid nitrogen	Freezing *via* isopentane precooled with dry ice	Freezing *via* liquid nitrogen vapor
pieces in		tubes pure/cryomolds with OCT	tubes pure/cryomolds with OCT	tubes pure/cryomolds with OCT
Mouse 1	liver	2/2	2/2	2/2
	muscle	2/2	2/2	2/2
Mouse 2	liver	2/2	2/2	2/2
	muscle	2/2	2/2	2/2
Mouse 3	liver	2/2	2/2	2/2
	muscle	2/2	2/2	2/2
	total liver	6/6	6/6	6/6
	total muscle	6/6	6/6	6/6
Study protocol		Investigation	Samples	Assessment criteria
1. Method optimization		Morphological evaluation of H&E-stains	All (36 liver +36 muscle samples)	Evaluable areas >50%
2. Method validation of freezing protocol with best morphological outcome		Morphological evaluation of HE-stains, RNA purity (A260nm/A280), RNA integrity number (RIN)	6 liver +6 muscle samples	Reproducibility and robustness
3. Method performance		Gene expression analyzes	All (36 liver +36 muscle samples)	No significant differences

In the second part, the validation part, the optimal freezing protocol was thus validated for reproducibility and robustness regarding evaluability of morphological criteria and quality of extracted RNA (purity, integrity). Finally, in the third part, performance testing was carried out by gene expression analyzes of defined biomarkers, selected based on tissue specificity.

Methodology acceptance criteria were 1) evaluable area of H&E-stains ≥50%, 2) A260 nm/A280 nm ratios ≥2.0, and 3) RIN values ≥ 7.0. Minimal values were considered for evaluable areas on H&E stains and mean values of corresponding samples for RNA purity and quality. Reproducibility was evaluated between samples from the same collection site with a coefficient of variation (CV) acceptance criteria of ≤25% for morphological evaluability and RNA integrity and ≤10% for RNA purity.

Robustness was assessed by investigating two different kinds of tissues (muscle and liver) from three individuals processed on three consecutive days with and without the use of OCT. Acceptance criteria were: 1) non-significant differences of morphological integrity and RNA purity for all tested tissue types with and without OCT. 2) non-significant differences of RIN values for one tissue type.

For performance testing the acceptance criterion was: non-significant differences in the normalized expression values (ΔCq) from RT-qPCR analysis of RNAs extracted from tissue samples that were frozen *via* the three here described freezing techniques.

### Sample collection protocol

Tissue samples were snap frozen either enclosed in cryo tubes (Sarstedt) or embedded in OCT in cryomolds (Sakura) (Table 1). All samples were then stored in the gas phase of liquid nitrogen.

### Cryosection/H&E staining/morphological evaluation

**FIGURE 1:**
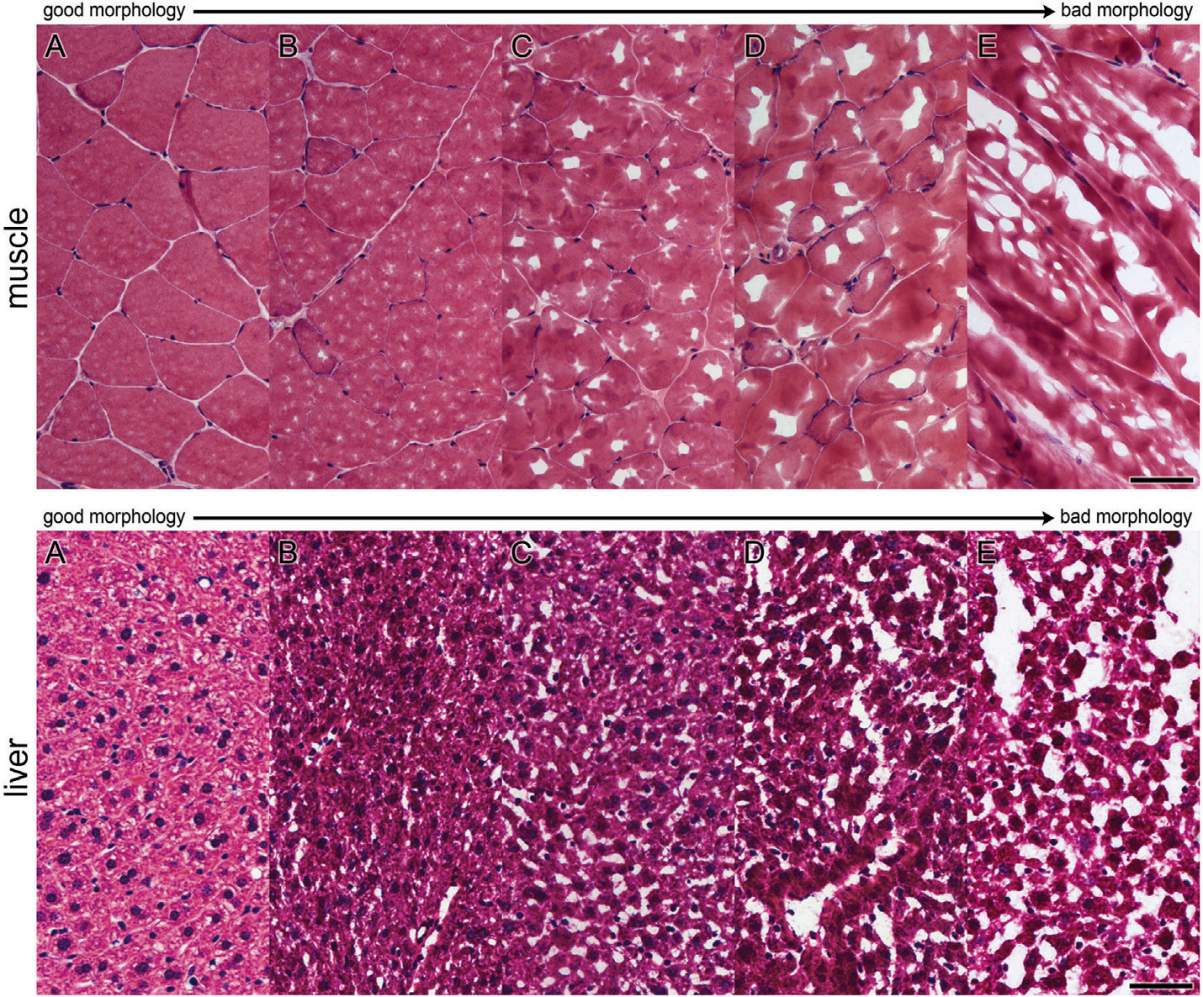
Evaluation scheme for assessment of morphological quality of areas. Morphology was evaluated on H&E-stained cryosections. Single areas were assigned to grading criteria shown in pictures **(A–E)** for muscle and liver tissue **(A)**: very good, **(B)** good, **(C)** acceptable, **(D)** bad, **(E)** very bad) [Bar **(A–E)**: 50 µm].

Seven µm thick cryosections were cut at a cryostat (CryoStar NX70, Thermo Scientific) and, after drying at room temperature, the tissue was stained with H&E (Romeis et al., 1989). Digitalization of the stained tissue was performed with an Aperio ScanScope Slidescanner (Leica Biosystems, Wetzlar, Germany). Tissue morphology was evaluated on these digitalized images and staged manually (Figure 1) with the Aperio ImageScope Software (Leica Biosystems, Wetzlar, Germany), according to criteria depicted above. The staged areas were measured and related to the total tissue area (given in %).

### RNA extraction/RNA-measurement

• RNA was extracted with the RNeasy Micro Kit (Qiagen). Cryosections were collected directly in tubes containing 1.4 mm ceramic beads for later disruption. Samples with OCT were rinsed twice with 1 ml precooled nuclease-free water to remove excess OCT. Then, lysis buffer was added, and samples were homogenized with the MagNA Lyser Instrument (Roche) for 15 s at 6000 rpm. After centrifugation, the supernatant was transferred to a new tube and RNA was extracted according to the manufacturer´s instructions (protocol for microdissected samples) including the optional DNAse treatment. RNA was eluted in 14 µl nuclease-free water and kept on ice for immediate quality control. The quality of isolated RNA was assessed regarding purity (absence of protein and other contaminants) and integrity (absence of degradation). RNA yield and purity were analyzed with a spectrophotometer (NanoDrop 2000c; Thermo Scientific) according to the manufacturer´s instructions. RNA purity was determined spectrophotometrically by measuring the A260/A280 nm absorbance ratio. Extracted RNA samples displaying a ratio of ≥2.0 are considered to possess “pure” RNA (Manchester, 1995).
• The RNA integrity number (RIN) was assessed by running RNA 6000 NanoChips on an Agilent Bioanalyzer 2100, according to the manufacturer´s instructions. Our method used for RNA quality was assessed to be “accurate” during the ISBER RNA Proficiency Testing Program. RIN values ≥7.0 are considered to be suitable for further analyzes according to previous publications (Jeffries et al., 2014; Zheng et al., 2019).

### Reverse transcription quantitative PCR

Three tissue-specific biomarkers: albumin (*Alb*) and apolipoprotein H (*Apoh*) for liver, and creatine kinase, muscle-type (*Ckm*) for muscle were selected for reverse transcription quantitative PCR (RT-qPCR). Primers were designed with the PrimerQuest primer design tool (https://www.idtdna.com/PrimerQuest/; Integrated DNA Technologies) or taken from literature (Day et al., 2018). Assays were validated by the generation of standard curves to calculated PCR reaction efficiencies. Assay details are listed in Supplemental file 1. Reverse transcription of 300 ng RNA was done with the High Capacity cDNA Reverse Transcription Kit (Thermo Fisher) according to the manufacturer’s protocol. RT-qPCR was performed in a 20 µl reaction volume containing 1x HOT FIREPol EvaGreen qPCR Mix Plus ROX (Solis BioDyne), 200 nM of each primer and 20 ng cDNA. All samples were analyzed in triplicates on a AriaMx Real-time PCR system (Agilent) with the following temperature protocol: initial activation at 95°C for 12 min, 40 cycles of 95°C for 15 s and 60°C for 1 min, followed by a melting curve analysis step (60–95°C). Two reference genes (RGs), β-actin (*Actb*) and eukaryotic translation initiation factor 2A (*Eif2a*) were included for normalization (Day et al., 2018). The expression stability of both RGs was assessed with the RefFinder tool (Xie et al., 2012), identifying *Eif2a* as the more stably expressed gene in both tissues. The efficiency-corrected target gene Cq values were normalized to *Eif2a* using the ΔCq approach (Silver et al., 2006). Mean Cq values obtained by RT-qPCR are enclosed in Supplemental file 2.

### Statistical analyzes

Mean, SD and CV% were calculated using Microsoft Excel. Significance was calculated with two-tailed Mann-Whitney *U* tests (GraphPad Prism, version 5.04) and a 5% significance threshold.

## Results

### Part one: Optimization

Three different protocols of snap-freezing with and without the use of OCT were applied and assessed in terms of good evaluability of morphological criteria on H&E stained tissue sections. The mean, the standard deviation and the minimum and maximum proportions of evaluable areas as a percentage of total areas are shown in Table 2. Minimal values of evaluable areas were below 50% for freezing in liquid nitrogen *via* isopentane and freezing in liquid nitrogen vapor. Only H&E stained sections of all samples directly frozen in liquid nitrogen met the defined acceptance criteria of ≥50% evaluable area. This finding based on minimal values agreed well with the calculated mean values, showing that no outlier values were used for evaluation. Based on the results, we considered direct freezing in liquid nitrogen to be the most appropriate method for subsequent validation.

**TABLE 2:** Optimization: Range of obtained results for evaluable areas in percent per freezing method.

Freezing method	Liver			Muscle		
	Mean/SD	Minimal-maximal values in % evaluable areas, acceptance criteria >50%		Mean/SD	Minimal-maximal values in % evaluable areas, acceptance criteria >50%	
Liquid nitrogen	99/1.7	96–100	passed	96/7.1	82–100	passed
Liquid nitrogen with OCT	99.8/0.4	99–100	passed	100/0	100–100	passed
Isopentane precooled with dry ice	85/22.0	45–100	failed	56/27.2	17–83	failed
Isopentane precooled with dry ice with OCT	99/1.0	98–100	passed	56/32.1	19–97	failed
Liquid nitrogen vapor (FluidX CryoPod^TM^-Carrier)	93/5.3	85–100	passed	66/18.4	49–89	failed
Liquid nitrogen vapor (FluidX CryoPod ^TM^-Carrier) with OCT	94/9.8	75–100	passed	64/22.5	39–98	failed

SD, standard deviation of the mean; CV, coefficient of variation; OCT, optimal cutting temperature compound.

### Part two: Validation

**FIGURE 2:**
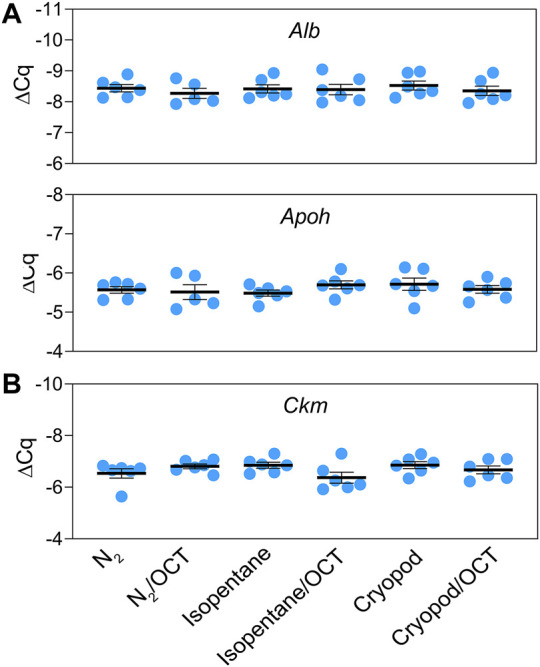
Relative mRNA expression levels (mean ± standard error) of the tissue-specific biomarkers: albumin and apolipoprotein H in liver **(A)**, and creatine kinase in muscle tissue **(B)**.

1) Reproducibility and robustness of good evaluability of morphological characteristics: Reproducibility was assessed in terms of percentage of evaluable areas on H&E-stains from samples from one collection site, individual for each mouse as well as together for all three mice. Direct freezing in liquid nitrogen led to constantly reproducible proportions of evaluable areas, showing intra-and interspecies CVs of ≤25% (Table 3). Common variations including the sampling of different tissue types and the use or disuse of OCT were analyzed in order to demonstrate the robustness of the freezing protocol. Obtained results showed no significant differences between the use and disuse of OCT tested for liver and muscle samples (*p* > 0.05).
2) Reproducibility and robustness of RNA purity and integrity: Direct freezing in liquid nitrogen provides reproducible results regarding RNA purity and integrity, displaying intra- and interspecies CVs of ≤10% and ≤25%, respectively (Table 4). All obtained results met the defined acceptance criteria of A260 nm/A280 nm ratios ≥2.0 and RIN values ≥ 7.0. For evaluation of the robustness, the impact of OCT on two tissue types was analyzed. Results are presented in Table 4. Extracted RNA from all liver and muscle samples with and without OCT met the defined criteria of ≥2.0 for A260 nm/A280 nm ratios (ratios ranged from 2.05 to 2.14) and ≥7.0 for RIN values (RIN ranged from 7 to 10). No significant differences were detected for RNA purity for all tissue types with and without OCT. However, the second acceptance criteria of robustness, non-significant differences of RIN values between the use and disuse of OCT, could not be met for both tissue types. While no differences were observed between RIN values of muscle and muscle + OCT samples (*p* > 0.05), significant differences were obtained for the comparison of RIN values from liver and liver + OCT samples (*p* < 0.05). It was obvious, that in liver samples frozen in combination with OCT, RIN values were lowered.
3) Performance testing by gene expression analyzes: The mRNA expression levels of the selected liver- (*Alb*, *Apoh*) and muscle (*Ckm*) biomarkers were evident in the respective tissues (Figure 2). No significant differences in gene expression were found between the freezing methods.

**TABLE 3:** Reproducibility and Robustness: Assessment of morphological evaluability of liver and muscle samples direct frozen in liquid nitrogen.

	Value	Mean/SD (One collection site, one individual)	CV% (One collection site, one individuals)	CV% (One collection site, three individuals)	Acceptance criteria <25%	With/without OCT
Mouse 1/liver	95.72	96.88/1.63	1.69	1.73	passed	*p* = 0.2530
	98.03					
Mouse 2/liver	99.29	99.65/0.50	0.50		passed	
	100					
Mouse 3/liver	100	100/0	0		passed	
	100					
Mouse 1/liver + OCT	99.07	99.55/0.66	0.66	0.42	passed	
	100					
Mouse 2/liver + OCT	100	100/0	0		passed	
	100					
Mouse 3/liver + OCT	100	100/0	0		passed	
	100					
Mouse 1/muscle	95.72	96.88/0.66	1.69	7.43	passed	*p* = 0.0740
	98.03					
Mouse 2/muscle	81.80	90.90/12.87	14.16		passed	
	100					
Mouse 3/muscle	100	100/0	0		passed	
	100					
Mouse 1/muscle + OCT	100	100/0	0	0	passed	
	100					
Mouse 2/muscle + OCT	100	100/0	0		passed	
	100					
Mouse 3/muscle + OCT	100	100/0	0		passed	
	100					

SD, standard deviation; CV, coefficient of variation; OCT, optimal cutting temperature compound.

**TABLE 4:** Reproducibility and Robustness: Assessment of RNA purity and RNA integrity of liver and muscle samples direct frozen in liquid nitrogen.

	RNA purity (A260nm/A280 nm)					RNA integrity number (RIN)				
	Mean/SD	CV% per mouse	CV% 3 mice	Acceptance criteria repro-ducibility <10%	Acceptance criteria robustness *p* > 0.05	Mean/SD	CV% per mouse	CV% 3 mice	Acceptance criteria repro-ducibility <25%	Acceptance criteria robustness *p* > 0.05
Mouse 1/liver	2.09/0.1	4.74	3.01	passed	Liver w/o OCT *p* = 0.5170 passed	8.75/0.49	5.66	6.48	passed	Liver w/o OCT *p* = 0.0222 rejected
Mouse 2/liver	2.14/0.1	3.97				9.40/0.42	4.51			
Mouse 3/liver	2.10/0.01	0.34				8.95/0.92	10.27			
Mouse 1/liver + OCT	2.10/0.01	0.34	0.73	passed	Liver w/o OCT *p* = 0.4192 passed	7.25/0.49	6.83	9.44	passed	
Mouse 2/liver + OCT	2.08/0.02	1.02				7.85/1.06	13.51			
Mouse 3/liver + OCT	2.08/-*	-*				7.0/-*	-*			
Mouse 1/muscle	2.09/0.01	0.68	1.44	passed	Muscle w/o OCT *p* = 0.8723 passed	9.45/0.78	8.23	6.18	passed	Muscle w/o OCT *p* = 0.7976 passed
Mouse 2/muscle	2.10/0.03	1.35				9.40/0.85	9.03			
Mouse 3/muscle	2.05/0.01	0.35				10/0	0			
Mouse 1/muscle + OCT	2.05/0.04	1.73	2.52	passed	Liver with OCT vs muscle with OCT *p* = 0.9271 passed	9.50/0.57	5.95	3.80	passed	
Mouse 2/muscle + OCT	2.14/0.01	0				9.70/0.42	4.37			
Mouse 3/muscle + OCT	2.08/0.06	3.07				9.45/0.78	1.43			

SD, standard deviation of the mean; CV, coefficient of variation; OCT, Optimal Cutting Temperature compound; *Data was excluded from evaluation, as the OCT block broke during the sample processing.

## Discussion

The use of isopentane as cryoconductor to speed up the freezing process of tissue samples has become standard for most pathology and research departments, when superb morphology is needed for evaluation. The superior suitability of this method in comparison to freezing in liquid nitrogen was supported by results from Steu et al. (2008) and Meng et al. (2014), showing better imaging of morphological characteristics when using isopentane. However, differences were moderate and dependent on the tissue type. In contrast, Kap et al. (2015) found no differences in morphology between the two snap-freezing techniques, investigating porcine liver samples.

These results raised the question, why most guidelines for biobanking adopted the freezing procedure with isopentane. Kap et al. (2015) even pointed out the possibility for biobanks to stop the use of isopentane after internal validation. Freezing with isopentane delivers slightly better morphological imaging, which might not be needed, if samples are used e.g., for molecular investigations. In such cases, freezing in liquid nitrogen will provide acceptable outcomes for morphological assessment and additional efforts of using isopentane are not justified. The usage of isopentane is time-consuming, inconvenient and includes additional risks of handling chemically hazardous substances and cross contamination. There are different procedures in place for snap-freezing tissue samples *via* isopentane. Specimens are frozen directly (Mager et al., 2007; ISO 20184, 2018; Doran et al., 2010) or enclosed e.g., in cryovials or histokinette cassettes (Mendy et al., 2017; ISO 20184, 2018; Australasian Biospecimen Network, 2007; Canadian Tissue Repository Network, 2020; NCI, 2016) and with (Australasian Biospecimen Network, 2007; Mager et al., 2007; NCI, 2016; Mendy et al., 2017) and without OCT (Mendy et al., 2017; Mager et al., 2007; ISO 20184, 2018; Australasian Biospecimen Network, 2007; ISBER, 2018; Canadian Tissue Repository Network, 2020; NCI, 2016). Isopentane can be precooled by liquid nitrogen (Mendy et al., 2017; Mager et al., 2007; ISO 20184, 2018; Australasian Biospecimen Network, 2007; ISBER Best Practices, 2018; NCI Best Practices, 2016), by dry ice (Mager et al., 2007; ISO 20184, 2018; ISBER Best Practices, 2018; NCI Best Practices, 2016) or by a −80°C freezer (ISO 20184, 2018; NCI Best Practices, 2016). There are no comparability studies showing whether those differences result in different morphological outcomes. It can be assumed, that processing in isopentane precooled with liquid nitrogen (−196°C), dry ice (−78.5°C) or a −80°C freezer leads to different freezing speeds. Muscle tissue is known to be sensitive to freezing delays by producing severe freezing artefacts. For best morphological results, muscle samples are immersed in isopentane, precisely at the moment, when solid white pebbles start to form, as then the optimal freezing temperature of −140°C to −149°C is reached (Meng et al., 2014). Consequently, freezing *via* isopentane cooled with liquid nitrogen at the exact suitable temperature range, seems to provide best morphological results.

In our study, we compared freezing in liquid nitrogen versus freezing *via* isopentane precooled with dry ice versus freezing in liquid nitrogen vapor. We observed best morphological outcomes for samples, which were frozen in liquid nitrogen. This was unexpected, but can be explained by details of the performed freezing protocols regarding freezing temperature, contact to freezing medium and sample size.

We precooled isopentane with dry ice, because this method is the most convenient for biobank routine use. It enables continuous working without interruptions for thawing of frozen isopentane when temperatures below the freezing point have been reached. However, isopentane cooled with dry ice does not reach the optimal freezing range of −140°C to −149°C and might therefore not represent the best way of isopentane application, which may also explain the poorer results obtained with this technique. It was evident, that the morphological integrity improves with decreasing freezing temperatures (isopentane cooled with dry ice: -80°C > liquid nitrogen vapor phase: -150 to −178°C > liquid nitrogen: -196°C).

Additionally, we enclosed all samples by either cryovials or OCT, which is, in our view, necessary when samples are intended for molecular investigations to avoid cross contamination. Therefore, direct freezing in isopentane and the change of isopentane when tissue sediment has settled at the bottom of the tube, as described (ISO 20184, 2018), is no option.

However, enclosing specimens prevents direct contact between specimen and isopentane or liquid nitrogen vapor, which may have affected the speed of the freezing process. Freezing of samples in liquid nitrogen that are already placed in vials, are reported to be particularly adversely affected by the Leidenfrost effect (Baker et al., 2013).

The sample size also affects the speed of the freezing process. We chose very small sample sizes of <0.3 cm per dimension, which may have allowed rapid freezing without the use of isopentane. This assumption is supported by Lee et al. (2020), who directly froze human muscle biopsies (0.3 cm × 2 cm) in liquid nitrogen without using isopentane and obtained high-quality cryopreserved samples suitable for histological analysis. Our optimal freezing protocol, freezing in liquid nitrogen, showed good reproducibility for morphological and molecular features. All defined acceptance criteria demonstrating the ability of the processing method to produce equally good sample material were met. Acceptance criteria for CVs for morphological evaluability and RNA integrity were chosen to be ≤ 25%, following recommendations of the Analytical Best Practices, (2016) guideline for analytical methods.

For RNA purity, we defined a CV ≤ 10% based on previously published data on the measurement method and on validation of DNA assays (Aranda et al., 2009; Mathay et al., 2016).

For assessment of robustness, we investigated two factors that were most likely to change during our biobanking working routine, tissue type and the use or disuse of OCT.

For testing different tissue types, we chose liver and muscle, representing completely different biological features. Liver tissue displays a very homogenous morphology and is considered less prone to form freezing artifacts. However, liver contains high levels of RNAse activity (ThermoFisher Scientific), which makes it difficult to extract high quality RNA. In contrast, muscle tissue is known to be sensitive to freezing artifacts initiated by suboptimal freezing conditions. Despite that, muscle tissue provides an environment that enables the extraction of high quality RNA (Bahar et al., 2007).

Results obtained for morphological integrity reflected the greater sensitivity of muscle tissue to the freezing process (Table 2).

OCT prevents tissues from the freezer-burn effect caused by liquid nitrogen, its positive impact should be particularly apparent for freezing in liquid nitrogen. Meng et al. (2014) reported that muscle tissues after direct freezing in liquid nitrogen showed significant freezing artefacts near surfaces, while internal areas exhibited good morphology. Consequently, it might be assumed, that the use of OCT will reduce artefacts of surfaces. However, for muscle tissue it is known, that OCT increases the formation of ice crystals (Meng et al., 2014). In our study, the use of OCT improved morphological integrity of muscle tissue after freezing in liquid nitrogen, no formation of artefacts could be observed. This shows that it is necessary to test in advance, whether the use of OCT improves or hinders the intended application, independent if morphological or molecular analysis are going to be performed. As an example, study results by Turbett and Sellner (1997) indicated, that OCT inhibits PCR analysis, while Steu et al. (2008) found no negative effect on PCR performance when using a column-based extraction method. Additionally, it was demonstrated, that OCT did not adversely influence RNA quality. In contrast, results of our study revealed that the use of OCT did adversely affect RNA quality of liver samples. This phenomenon could not be observed for muscle tissues and was independent from the performed freezing techniques (see Supplemental file 3). To the best knowledge of the authors, an adverse effect of OCT on RIN values of liver samples has not been reported before. Tissue samples embedded with OCT were washed with sterile water to remove the OCT completely to enable RNA extraction by columns. Of course, this washing step may be the cause for RIN decrease. However, both tissue types were treated identically, but muscle tissue did not show any degradation of RNA. This may be due to the high stability of RNA isolated from muscle tissues (Bahar et al., 2007).

RT-qPCR was used to investigate the eligibility of the different freezing methods for subsequent mRNA quantification. Comparable expression levels of *Alb* and *Apoh* in liver, and *Ckm* in muscle were detected in all samples independent of the freezing process. Our results clearly showed that all tested freezing methods are equally suitable for down-stream RNA analyzes.

Data presented here demonstrates that our standard biobank freezing protocol, freezing of enclosed tissue samples in liquid nitrogen with and without OCT, is fit for biobank purposes, even if the applied freezing protocol does not fully comply with standard biobanking guidelines. Our protocol showed good reproducibility and robustness in terms of morphological evaluability, RNA 260/280 nm ratios and RIN values. Only one acceptance criterion could not be met. RIN values for liver samples embedded in OCT displayed significantly lower RIN values than liver samples without OCT. As the decrease in RIN values for liver samples with OCT turned out to be a general feature independent from the applied freezing protocol, our freezing protocol was rated to be robust. However, further tissue types have to be included for assessment of RIN values to investigate tissue-specific effects. Performed gene expression analyzes showed no significant differences in relation to the freezing protocols applied. Therefore, it can be concluded that the standard freezing protocol of the Vetbiobank (Walter et al., 2020) is suitable to generate samples for morphological evaluations as well as for down-stream RNA analyzes.

## Data Availability

Datasets used and/or analyzed in this study are available from the corresponding author upon reasonable request.
