# Supplementary material for: Example for process validation in biobanking: Fit for purpose testing of a cryopreservation method without isopentane
Source: Front Mol Biosci. 2022 Sep 30;9:876670. doi: 10.3389/fmolb.2022.876670 (PMC9562646; doi:10.3389/fmolb.2022.876670)
Supplement: Supplementary file 2 [file Table1.DOCX]

**RT-qPCR primer**

| **NCBI accession number*** | **Gene name** | **Primer sequence (5‘ – 3‘)** | **Amplicon size (bp)** | ***E* (%)** | **R^2^** | **Reference** |
| --- | --- | --- | --- | --- | --- | --- |
| NM_007393.5 | Actin, beta | F: CTAAGGCCAACCGTGAAAAG  R: ACCAGAGGCATACAGGGACA | 104 | 91.5 | 0.996 | Day et al., 2018 |
| NM_009654.4 | Albumin | F: CACACTTCCAGAGAAGGAGAAG  R: CTTCAGTTGCTCCGCTGTA | 94 | 92.4 | 1 | - |
| NM_013475.4 | Apolipoprotein H | F: GCCCAGATATTCCTGCTTGT  R: AGAGTTGTTCCCAGCTGAAG | 101 | 90.2 | 0.994 | - |
| NM_007710.2 | Creatine kinase, muscle | F: GTGTCACCTCTGCTGCTG  R: CGTTCACCCACACAAGGAA | 100 | 100.4 | 0.994 | - |
| NM_001005509.2 | Eukaryotic translation initiation factor 2A | F: ACTTTTAGTAAGGATGGGACATTGTT  R: TCCCTTGTTAGCGACATTGA | 78 | 91.3 | 0.998 | Day et al., 2018 |

*National Center for Biotechnology Information (NCBI), Entrez Gene (https://www.ncbi.nlm.nih.gov/gene); F and R: forward and reverse primer; *E*: PCR amplification efficiency; R^2^: correlation coefficient of standard curve
