# Supplementary material for: Example for process validation in biobanking: Fit for purpose testing of a cryopreservation method without isopentane
Source: Front Mol Biosci. 2022 Sep 30;9:876670. doi: 10.3389/fmolb.2022.876670 (PMC9562646; doi:10.3389/fmolb.2022.876670)
Supplement: Supplementary file 3 [file Table3.docx]

Supplemental File 3. Summary of RNA integrity numbers of liver and muscle samples applying 3 different freezing methods.

**RNA integrity number (RIN)**

1. liquid nitrogen (2) freezing via isopentane (3) freezing in the vapor of liquid nitrogen precooled on dry ice [FluidX (now Brooks Life Science) CryoPod™]

Mouse 1/liver 9.1 9.6 9.1

8.4 9.1 9.5

Mouse 2/liver 9.7 8.8 9.9

9.1 9.1 9.9

Mouse 3/liver 8.3 9.4 9.2

9.6 9.7 9.4

Results after two tailed Mann Whitney test:

Method (1) liver w/o OCT, p<0.05 →not robust

muscle w/o OCT, p>0.05 →robust

Method (2) liver w/o OCT, p<0.05 →not robust

muscle w/o OCT, p>0.05 →robust

Method (3) liver w/o OCT, p<0.05 →not robust

muscle w/o OCT, p>0.05 →robust

muscle w/o OCT
p=0.7976
passed

Mouse 1/liver + OCT 6.9 8.4 7.4

7.6 6.9 7.8

Mouse 2/liver + OCT 8.6 6.5 8.2

7.1 7.4 7.3

Mouse 3/liver + OCT -* 8.5 6.9

7 8.4 6.4

Mouse 1/muscle 10 -* 9.3

8.9 -* 9.7

Mouse 2/muscle 10 10 -*

8.8 10 10

Mouse 3/muscle 10 10 10

10 9.4 10

Mouse 1/muscle + OCT 9.1 8.1 9.9

9.9 8.3 10

Mouse 2/muscle + OCT 9.4 7.9 10

10 8.2 10

Mouse 3/muscle + OCT 10 5.7 10

9.8 7.1 10

OCT, Optimal Cutting Temperature compound; *data was excluded from evaluation, as the OCT block broke during the sample processing.
